# Arabidopsis response to the spider mite *Tetranychus urticae* depends on the regulation of reactive oxygen species homeostasis

**DOI:** 10.1038/s41598-018-27904-1

**Published:** 2018-06-21

**Authors:** M. Estrella Santamaría, Ana Arnaiz, Blanca Velasco-Arroyo, Vojislava Grbic, Isabel Diaz, Manuel Martinez

**Affiliations:** 1 Centro de Biotecnología y Genómica de Plantas, Universidad Politécnica de Madrid (UPM) - Instituto Nacional de Investigación y Tecnología Agraria y Alimentaria (INIA), Campus Montegancedo UPM, 28223 Pozuelo de Alarcón, Madrid, Spain; 2 Departamento de Biotecnología-Biología Vegetal, Escuela Técnica Superior de Ingeniería Agronómica, Alimentaria y de Biosistemas, UPM, 28040 Madrid, Spain; 3 Department of Biology, The University of Western Ontario, 1151 Richmond St, London, ON N6A 5B7 Canada

## Abstract

Reactive oxygen species (ROS) are molecules that play a prominent role in plant response to numerous stresses, including plant interactions with herbivores. Previous findings indicate that Arabidopsis plants showed an increase in H_2_O_2_ accumulation after *Tetranychus urticae* infestation. Despite its importance, no information has been reported on the relationships between ROS-metabolizing systems and the spider mite-triggered plant-induced responses. In this work, four ROS-related genes that were differentially expressed between the resistant Bla-2 and the susceptible Kon Arabidopsis accessions were selected for the analysis. These genes encode proteins putatively involved in the generation (BBE22) and degradation (GPX7 and GSTU4) of H_2_O_2_, and in the degradation of ascorbate (AO). Overexpressing *BBE*22 and silencing *GPX7*, *GSTU4* and *AO* resulted in higher leaf damage and better mite performance relative to the wild-type plants. Minor effects on H_2_O_2_ accumulation obscure major effects on the expression of genes related to ROS-metabolism and JA and SA signaling pathways, and on ROS-related enzymatic activities. In conclusion, the integration of ROS and ROS-related compounds and enzymes in the response of Arabidopsis to the spider mite *T*. *urticae* was confirmed. However, the complex network involved in ROS signaling makes difficult to predict the impact of a specific genetic manipulation.

## Introduction

Plants, as sessile organisms, have to cope with hostile conditions derived from the attack of other organisms. Plants rely on a battery of mechanisms to detect herbivores in order to respond with specific defenses. Plant defenses are triggered when specific receptors detect either Herbivore-Associated Molecular Patterns (HAMPs), Microbial-Associated Molecular Patterns (MAMPs) from the herbivore-associated gut endosymbionts, Damage-Associated Molecular Patterns (DAMPs) that results from the damage incurred by plant tissues as a consequence of herbivore feeding, or the presence of volatiles emitted as plant-to-plant signaling^1–3^.

Early events in plant-herbivore interactions commence with membrane potential depolarization at the feeding site, alteration in cell membrane and ion imbalance followed by changes in the intracellular Ca^2+^ and generation of the reactive oxygen and/or nitrogen species (ROS/RNS)^4,5^. A cascade of protein kinases as calcium-sensor proteins lead to the synthesis of phytohormones and the activation of transcription factors that regulate the expression of herbivore-responsive genes^6–8^. Host transcriptomic, proteomic and metabolomic profiles after arthropod feeding, oviposition or application of insect secretions have demonstrated that plants may discriminate between herbivores to activate herbivore-specific plant responses^9–12^. These responses are regulated by a complex hormonal cross-talk centered at Jasmonic (JA) and Salicylic (SA) acids^13–15^.

As ROS levels, especially H_2_O_2_, increase during herbivore feeding or egg deposition, a dual role has been attributed to ROS molecules as direct defenses and as a part of oxidative signaling pathways in plants^16^. ROS may directly damage biological molecules such as nucleic acids, amino acids, proteins and lipids. A particularly damaging effect results from the onset of autocatalytic lipid peroxidation leading to loss of membrane integrity and cell death. ROS also act as signaling molecules with an important role in transmitting information to allow appropriate cellular responses to developmental and environmental changes^17^. To constrain the ROS-inflicted damage and to regulate the ROS-signaling pathways, plants have evolved multiple detoxification systems for efficient removal of H_2_O_2_ and phospholipid hydroperoxides. Compartmentation of the ROS processing systems including cellular components that interact with ROS and transmit oxidative signals (such as the ascorbate/glutathione system) are crucial to keep ROS below damaging levels, but to permit relaying ROS signals^18,19^. Accumulating evidence supports the effect of herbivore infestation on stress signaling networks triggered by ROS and redox-sensitive factors including hormone-signaling pathways^5,20^.

While ROS molecules are considered necessary to orchestrate the defense responses, their effect on plant-resistance/susceptibility to a particular herbivore depends on the specific plant-herbivore interaction. For example, the aphid *Myzus persicae* grew better when reared on potato leaves containing high ascorbate levels than on leaves with low ascorbate^21^. In contrast, Arabidopsis *vtc1-1* mutants (ascorbate deficient) were shown to be more susceptible to the lepidopteran pest *Spodoptera littoralis* than control lines^22^.

Among arthropod herbivores, phytophagous mites are important agricultural pests worldwide. The two-spotted spider mite *Tetranychus urticae* Koch (Acari: Tetranychidae) has a wide host range, feeding on more than 1,100 host plants including over 100 agricultural crops^23^. *T*. *urticae* pierces parenchymatic plant cells using a stylet to suck the cell content causing severe chlorosis^24^. The availability of *T*. *urticae* genome sequences^25^, the mite ability to feed on the model species *Arabidopsis thaliana*, and the existence of tools to analyze the Arabidopsis-mite interaction^26,27^, provided an outstanding opportunity for functional studies of their reciprocal responses. Several studies of Arabidopsis responses to spider mites have revealed the role of major hormonal pathways in host response^28,29^ and identified Arabidopsis toxic compounds^30,31^.

Previous findings indicate that the effect of the *Mite Attack Triggered Immunity* (*MATI*) gene on mite herbivory correlated with changes in the plant antioxidant status^28^. The levels of H_2_O_2_ and electrolytic leakage increased after mite infestation of leaves from the wild-type and silencing *mati* plants. This phenotype was associated with plant susceptibility to mite herbivory. In contrast, the infestation of *MATI* over-expressing plants lead a moderate accumulation of H_2_O_2_ without changes in cellular electrolytic leakage that were associated with a higher resistance to mite herbivory.

Despite its importance, the role of ROS-metabolizing systems in mite-induced plant responses is unknown. In this work, we have focused on four genes (*BBE*22, *GPX7*, *GSTU4* and *AO*) putatively involved in ROS homeostasis. These genes showed consistently higher expression levels in the resistant Bla-2 relative to susceptible Kon Arabidopsis accessions after mite feeding. The functional characterization of these genes supports a role for the ROS in the response of Arabidopsis to *T*. *urticae* through a complex network of ROS-related genes.

## Results

### Genes involved in ROS homeostasis are differentially expressed in response to spider mite feeding

We have previously highlighted the natural genetic variation of resistance between Arabidopsis accessions to *T*. *urticae*, identifying Bla-2 (resistant) and Kondara (susceptible) as accessions at the opposing ends of the spectrum^29^. Besides, we made a gene ontology enrichment analysis of the 1109 and 993 differential expressed genes between Bla-2 and Kon reported upon *T*. *urticae* infestation or in absence of spider mite herbivory, respectively^29^. Mining the dataset coming from these analyses, several functional categories related to ROS homeostasis appeared over represented in transcriptomic comparisons related to *T*. *urticae* infestation or to resistance/susceptibility of Arabidopsis accessions (Table 1). To further explore the defensive role of single genes directly related to ROS homeostasis, four genes differentially expressed in Bla-2 and Kon upon *T*. *urticae* infestation were selected (Table 2). These candidate genes were: *AT4G39830* (*AO*), *AT4G31870* (*GPX7*), *AT*2*G*2*9460* (*GSTU4*) and *AT4G*2*0860* (*BBE*22). The putative implication of these proteins in ROS homeostasis may be deduced from the schematic metabolic network regarding hydrogen peroxide production and elimination showed in Supplementary Fig. S1. BBE22 would be involved in the generation of H_2_O_2_ from reduced compounds; AO would have the role in the degradation of ascorbate to (mono)dehydroascorbate, and GPX7 and GSTU4 in the degradation of H_2_O_2_ to water using glutathione as electron donor. RT-qPCR analysis carried out in several Arabidopsis tissues showed that mRNA expression levels were detected in most of the tissues for the four genes, which include leaves and rosettes (Supplementary Fig. S2).

**Table 1:** ROS categories differentially represented under different comparisons related with *Tetranychus urticae* (TU) infestation or with resistance/susceptibility of accessions.

Comparison	Go Id	Term	Annotated	Significant	Expected	Classic fisher
1 h TU/Control	0010310	Regulation of H_2_0_2_ metabolism	187	31	6.2	1.2E-13
	0006979	Response to oxidative stress	600	43	19.91	2E-10
3–24 h TU/Control	0010310	Regulation of H_2_0_2_ metabolism	187	51	7.01	6.9E-30
	0055114	0xidation-reduction process	1521	90	57.01	9.4E-10
Bla-2 > Kon	0010310	Regulation of H_2_0_2_ metabolism	187	16	5.09	5.50E-05
Bla-2 > Kon, TU induced	0010310	Regulation of H_2_0_2_ metabolism	187	11	0.93	2.30E-09

**Table 2:** *In silico* description of selected ROS-related genes.

Gene Id	Name	Involved in	Domain
*AT4G39830*	Cupredoxin superfamily protein (*AO*)	Oxidation-reduction process (L-ascorbate oxidase activity)	L-ascorbate oxidase Multicopper oxidase
*AT4G31870*	Glutathione peroxidase 7 (*GPX7*)	Oxidation-reduction process, Response to karrikin, response to oxidative stress	Glutathione peroxidase Thioredoxin-like
*AT2G29460*	Glutathione S-transferase TAU 4 (*GSTU4*)	Glutathione metabolic process, response to toxic substance, toxin catabolic process	Glutathion S-transferase Thioredoxin-like
*AT4G20860*	FAD-binding Berberine family protein (*BBE22*)	Oxidation-reduction process	Berberine/berberine-like Oxygen oxidoreductase covalent FAD-binding site FAD linked oxidase, N-terminal, FAD-binding, type 2

**Figure 1:**
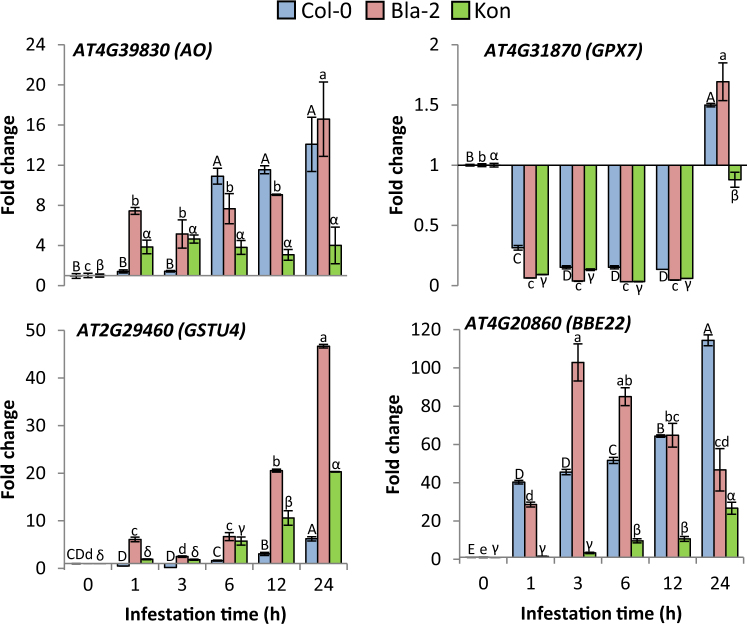
Gene expression in Arabidopsis Bla-2, Kon and Col-0 accessions in response to *T*. *urticae* infestation. The expression of *AT4G39830*, *AT4G31870*, *AT2G29460* and *AT4G20860* genes was quantified at 1, 3, 6, 12 and 24 h post-infestation by RT-qPCR assays. Gene expression referred as fold change (2^−ddCt^). Data are means ± SE of three replicates. Different letters indicate significant differences (P < 0.05, One-way ANOVA followed by Student-Newman-Keuls test).

To confirm the inclusion of these genes in Arabidopsis responses to *T*. *urticae* feeding and their differential induction in Arabidopsis accessions, we infested Col-0, Bla-2 and Kon plants with spider mites and monitored levels of *BBE*22, *AO*, *GPX7* and *GSTU4* using the RT-qPCR assays (Fig. 1). *BBE22* expression was induced from 1 h on post infestation in Bla-2 and Col-0, picking at 3 h in Bla-2 and 24 h in Col-0. In Kon, induction was observed from 6 h post infestation and reached lower values than in Bla-2 and Col-0 accessions. *AO* was induced from 1 h in Bla-2 and Kon. Expression levels remain stable in Kon but significantly increased in Bla-2 at 24 h. In Col-0, induction was observed from 6 h and reached similar values to that observed in Bla-2 at 24 h post infestation. *GSTU4* was induced earlier and at higher level in Bla-2 than in Col-0 and Kon. In contrast, *GPX7* was initially (1–12 h post mite infestation) repressed in all three Arabidopsis accessions but was induced at 24 h in Bla-2 and Col-0 and reached almost the basal level in Kon. Thus, all four genes (*BBE22*, *AO*, *GPX7* and *GSTU4*) are induced by *T*. *urticae* herbivory and their expression levels (and timing in the case of GSTU4) correlate with the Arabidopsis resistance to *T*. *urticae*.

### Characterization of T-DNA insertion lines in differentially expressed ROS related genes

**Figure 2:**
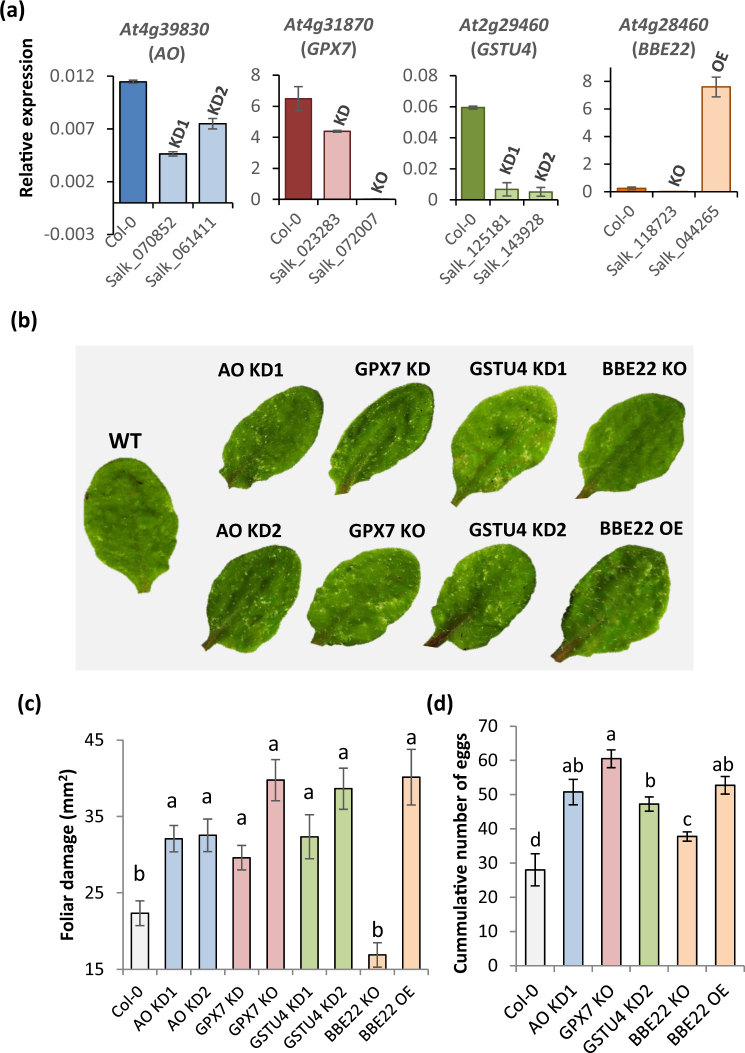
Plant damage and spider mite performance upon feeding on Col-0 and T-DNA insertion lines. (**a**) Relative expression values of the targeted genes in the T-DNA insertion lines. T-DNA lines for the same gene are represented in the same colour. Data are means ± SE from three replicates. (**b**) Visualization of leaf damage on Arabidopsis Col-0 and T-DNA insertion lines 4 days after *T*. *urticae* feeding. (**c**) Quantification of leaf damage on Arabidopsis Col-0 and T-DNA insertion lines 4 days after *T*. *urticae* feeding. Data are means ± SE from six replicates. Different letters indicate significant differences (P < 0.05, One-way ANOVA followed by Student-Newman-Keuls test). (**d**) *T*. *urticae* performance, referred as number of mite eggs. Data are means ± SE from eight replicates. Different letters indicate significant differences (P < 0.05, One-way ANOVA followed by Student-Newman-Keuls test).

To investigate the role of the candidate ROS homeostasis related-genes in plant defense against *T*. *urticae*, available T-DNA insertion lines for these genes were characterized. The location of the T-DNA insertion in each gene is shown in Supplementary Fig. S3. The expression levels of the targeted genes were quantified in homozygous lines (Fig. 2a). In the AO lines (Salk_070852 and Salk_061411), T-DNA insertions were in coding regions (with the exception of the isoform 4 of this gene, in which an insertion was in a putative intron). As the expression of the gene was not totally abolished, these lines were considered as knock-down lines (AO KD1 and AO KD2). The T-DNA insertions in the GPX7 lines were also in coding regions. Whereas the insertion of the T-DNA into exon 4 (Salk_023283 line) abolished the expression of *GPX7* (GPX7 KO, knock-out), the T-DNA insertion in exon 2 (Salk_072007 line) only resulted in a reduced *GPX7* expression (GPX7 KD, knock-down). For *GSTU4*, one T-DNA insertion was located in the second exon, at the end of the coding region, and the other inserted within the 3′UTR. In both lines, the expression of the gene was considerably reduced but still quantifiable. Thus, these lines were considered knock-down lines (Salk_1439280, GSTU4 KD1; Salk_125181, GSTU4 KD2). Finally, T-DNA insertions in the *BBE22* gene were located in the promoter region. Interestingly, these insertions had opposite effects. Whereas the insertion closest to the 5’UTR caused a complete lack of gene expression (Salk_118723, BBE22 KO), the T-DNA insertion further upstream resulted in a strong over expression of the gene (Salk_044265, BBE22 OE).

### Effects of ROS homeostasis related genes on plant resistance and mite performance

To investigate the role of the candidate ROS homeostasis related-genes in plant defense, T-DNA insertion lines and non-transformed Col-0 controls were infested with mites and plant damage (chlorotic area) was visualized and quantified four days after mite feeding (Fig. 2b,c). All T-DNA insertion lines for *AO*, *GPX7* and *GSTU4* genes showed significantly more damage than non-transformed Col-0 infested plants (among 1.4 and 1.8-fold difference depending on the line). In contrast, whereas the damage in the BBE22 KO line was quite similar to that observed in infested Col-0 WT plants, the damaged area in the BBE22 OE line was 1.8 fold greater than the damage quantified in Col-0 WT line. To ensure that damaged area correlated with mite performance, we determined mite fecundity after feeding on the WT and the T-DNA insertion lines. Synchronized females feeding on WT plants had significantly lower fecundity rates than those feeding on T-DNA insertion lines (Fig. 2d). Interestingly, with the exception of BBE22 KO plants, mite oviposition ratio correlated to plant damage. Thus, for most lines greater leaf damage reflects mite’s ability to feed more intensely resulting in greater fecundity, indicating that T-DNA insertions in ROS-related genes affected plant defences against spider mite herbivory.

### Effects of genetically modified ROS related genes on ROS homeostasis

**Figure 3:**
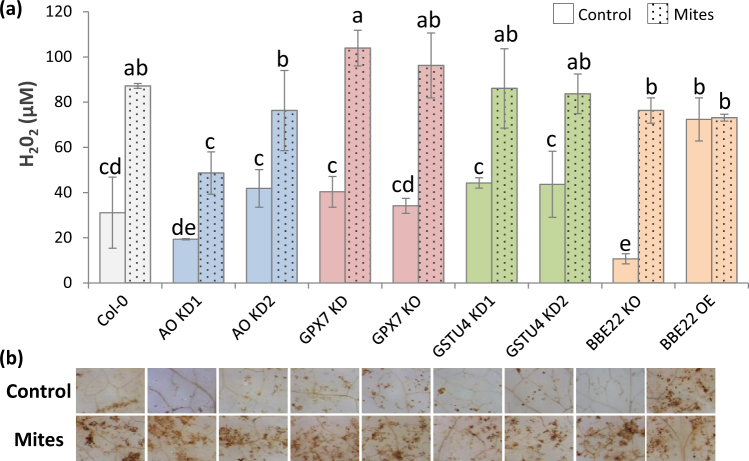
Accumulation of H_2_O_2_ in Col-0 and T-DNA insertion lines infested with *T*. *urticae*. (**a**) H_2_O_2_ quantification on Arabidopsis Col-0 and T-DNA insertion lines 24 h after *T*. *urticae* feeding. Data are means ± SE from three replicates. Different letters indicate significant differences (P < 0.05, Two-way ANOVA followed by Student-Newman-Keuls test). (**b**) H_2_O_2_ visualization in leaves by DAB staining after 24 h *T*. *urticae* feeding.

Alterations in the expression of ROS related genes were expected to affect the ROS homeostasis. To characterize these changes, we quantified the H_2_O_2_ accumulation in wild-type and mutant Arabidopsis plants. To determine if ROS homeostasis in these plants changes as a result of mite herbivory, a portion of these plants were infested by mites (Fig. 3a). Using the fluorescence-based assays we found that H_2_O_2_ levels were similar in WT Col-0 and majority of T-DNA lines. BBE22 lines had a different pattern of H_2_O_2_ accumulation. The basal H_2_O_2_ levels were significantly lower in the KO line and higher in the OE line relative to the WT Col-0 plants. As expected, the H_2_O_2_ levels increased in all plants upon spider mite infestation, with the exemption of the BBE22 OE plants that accumulated high levels of H_2_O_2_ in the absence of mite feeding. The accumulation of H_2_O_2_ upon spider mite infestation was corroborated by DAB staining visualization, which displayed slight variations in the stained signals (Fig. 3b).

**Figure 4:**
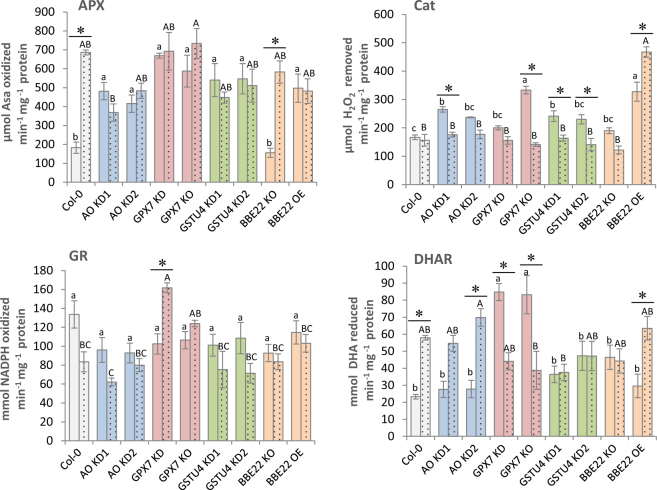
Activities of several enzymes involved in H_2_O_2_ metabolism in Col-0 and T-DNA insertion lines. Data from Col-0 and T-DNA insertion lines without mites (plain columns) and upon 24 hours infestation with *T*. *urticae* (dotted columns). APX (Ascorbate Peroxidase), Cat (Catalase), GR (Glutathione Reductase), DHAR (Dehydroascorbate Reductase), Asa (Ascorbic Acid), NADPH (nicotinamide-adenine dinucleotide phosphate), DHA (Dehydroascorbic Acid). Data represent means ± SE from at least six replicates. Different letters indicate significant differences among genotypes within control or mite infested lines (P < 0.05, One-Way ANOVA followed by Student-Newman-Keuls test) and asterisks indicate significant differences among control and infestation within each genotype (P < 0.05, t-student test).

Besides H_2_O_2_ accumulation, the activity of several enzymes involved in its metabolism was quantified (Fig. 4). Whereas catalase (Cat) and ascorbate peroxidase (APX) activities are directly related to H_2_O_2_ degradation, dehydroascorbate reductase (DHAR) and glutathione reductase (GR) are involved in the regeneration of ascorbate and reduced glutathione, compounds that can be used by ascorbate peroxidases or glutathione peroxidases and S-transferases to reduce H_2_O_2_ (Supplementary Fig. S1). The reduced expression of H_2_O_2_-synthesyzing enzyme BBE22 in BBE22 KO line did not affect activities of APX, Cat, GR or DHAR enzymes in presence or the absence of mite feeding. These enzymes had similar activities to these observed in the Col-0 WT plants (Fig. 4). The overexpression of *BBE22* in BBE22 OE line led to increased basal activity of APX and Cat that are directly related to H_2_O_2_ degradation. Mite feeding did not affect the APX activity, but led to the increased Cat activity in these lines relative to the WT. APX quantification showed a higher basal activity in all T-DNA lines with reduced expression of genes encoding enzymes involved in the H_2_O_2_ catabolism (*AO*, *GPX7* and *GSTU4*) relative to Col-0 WT. However, while the APX activity increased upon mite feeding in WT plants; it did not change further in AO, GPX7 and GSTU4 T-DNA lines. The increase in basal activity was also observed for the Cat activity in these mutant lines. After mite attack, catalase activity did not vary in Col-0 and was reduced to Col-0 levels in all T-DNA lines that disrupt genes acting in the H_2_O_2_ catabolism. Basal GR activity was similar for all lines. After mite infestation, this activity significantly increased only in GPX7 KO lines. Finally, GPX7 lines also showed higher basal DHAR activity while the rest of the lines had activity levels comparable to the WT plants. Whereas the activity after *T*. *urticae* feeding was reduced in the GPX7 KO lines, in WT Col-0 and AO KD the DHAR activity increased and it remained at the basal level in the GSTU4 KD lines. In summary, the overexpression of BBE22 that correlated with the increased generation of H_2_O_2_ (Fig. 3) resulted in the increased activity of APX and Cat enzymes that are directly related to H_2_O_2_ degradation. The alteration of genes encoding enzymes involved in H_2_O_2_ catabolism resulted in more complex changes in the enzymatic activities of APX, Cat, GR and DHAR, suggesting the existence of compensatory relationship among enzymes involved in H_2_O_2_ catabolism that is consistent with the overall unperturbed changes in levels of H_2_O_2_ in these mutant plants (Fig. 3).

**Figure 5:**
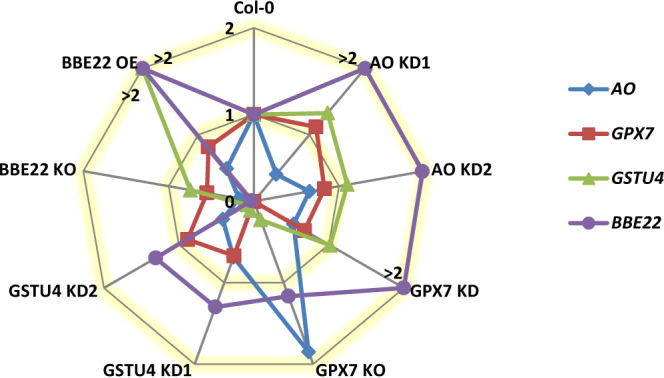
Expression changes in T-DNA insertion lines for selected ROS-related genes (*AO*, *GPX7*, *GSTU4* and *BBE22*). Gene expression as fold change (2^−ddCt^) referred to the expression in Col-0. Data means and SE are compiled in Supplementary Table 1. For clarity, fold changes higher than 2 are represented in the fold change 2 line and marked with a > symbol.

To test this assumption, the expression of *BBE22*, *AO*, *GPX7* and *GSTU4* was analyzed by RT-qPCR in the T-DNA insertion lines (Fig. 5 and Supplementary Table 1). *AO* expression was repressed in all T-DNA lines with the exception of GPX7 KO, in which its expression was induced. *GPX7* expression was repressed in one GSTU4 KD line and in the BBE22 KO line. *GSTU4* transcriptional levels were reduced in the GSTU4 KD lines and the GPX7 KO line and were increased in the BBE22 OE line. Finally, the expression of *BBE22* was induced in the for AO and GPX7 KD lines. Thus, the expression of any one of the tested genes involved in the ROS homeostasis is affected by the perturbation of the expression of other genes within this process.

### Effects of genetically modified ROS related genes on defense-related hormonal pathways

**Figure 6:**
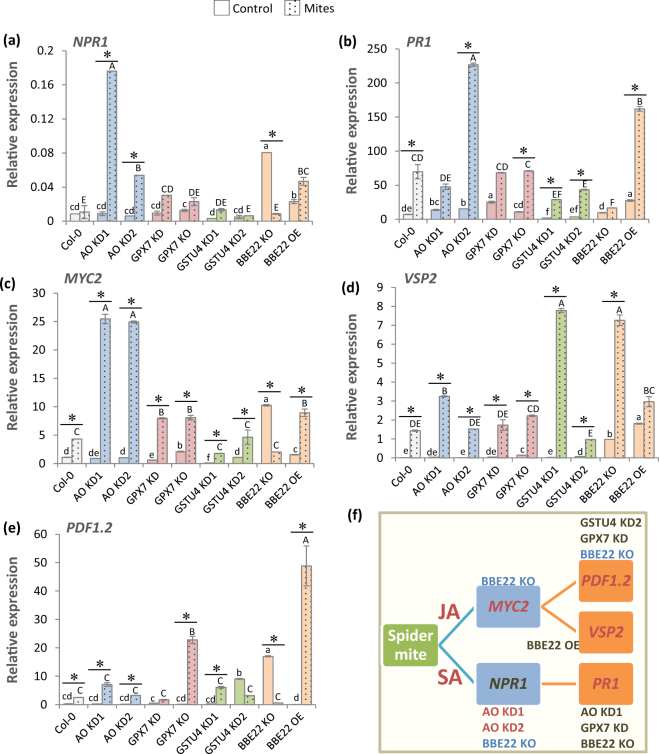
Relative expression values of hormone-related genes on Arabidopsis Col-0 and T-DNA insertion lines 24 h upon *T*. *urticae* feeding. (**a**) *NPR1*, Non-expresser of PR gene1. (**b**) *PR1*, Pathogenesis Related. (**c**) *MYC2*, MYC2 transcription factor. (**d**) *VSP2*, Vegetative Storage Protein 2. (**e**) *PDF1*.*2*, Plant Defensin. Gene expression levels were normalized to the ubiquitin gene expression. Data are means ± SE from three replicates. Different letters and asterisks indicate significant differences (P < 0.05, One-way ANOVA followed by Student-Newman-Keuls test). (**f**) Diagram showing the T-DNA lines with different trend of expression response to *T*. *urticae* than Col-0 WT plants for each analysed gene (red, induction; blue, repression; dark cinnamon, no change in trend).

Previously described effects of the perturbation in the expression of ROS related genes on mite-inflicted plant damage and mite performance suggest that these genes could also affect the signals that trigger hormone responsive pathways. As JA and SA are the main hormones involved in plant defense against spider mites, the expression of several genes controlled by these hormones was checked by RT-qPCR (Fig. 6). *Non-expresser of PR gene1* (*NPR1*) involved in SA-triggered transcriptional regulation and *Pathogenesis Related* (*PR1*) genes were chosen as markers of SA responses. Basal *NPR1* expression was quite similar in most lines. The BBE22 KO and OE lines had higher expression that was stronger in the BBE22 KO line (Fig. 6a). Upon mite feeding, the significant increase of *NPR1* expression was detected in AO KD and BBE22 OE lines. The basal *PR1* expression levels were elevated in lines with reduced activity of H_2_O_2_-catabolism related enzymes (Fig. 6b). Mite feeding strongly induced the expression of *PR1* in all lines but BBE22 KO, reaching the highest levels in AO KD2 and BBE22 OE lines. Further, we selected MYC2 transcription factor (*MYC2*), *Vegetative Storage Protein 2* (*VSP2*) and *Plant Defensin* (*PDF1*.*2*) as marker genes for the JA pathway. While *MYC2* mediates the JA-responses to activate the expression of *VSP2*, the expression of *PDF1*.*2* is dependent on both JA and ethylene (ET) signaling. In general, the expression of *MYC2*, *VSP2* and *PDF1*.*2* was induced by mite feeding (Fig. 6c–e). Consistent with the antagonistic relationship between the *VSP2* and *PDF1*.*2* pathways in plant defence against herbivory^32^, BBE22 KO and OE lines had opposing effects on the expression of *VSP2* and *PDF1*.*2* (Fig. 6d,e). While the reduced expression of *BBE22* led to the high expression of the *VSP2*, the *BBE22* overexpression favored the expression of the *PDF1*.*2* upon mite feeding. Overall, alterations in the expression of genes involved in the ROS homeostasis were reflected in an alteration of JA and SA signaling pathways in response to mite feeding (Fig. 6f).

## Discussion

Multiple genes encoding ROS-processing enzymes, including peroxidases, reductases, and dehydrogenases are present in plants. A key challenge is to elucidate the physiological importance of specific members in terms of their potential influence on biotic stress resistance programs. The comparison of differentially expressed genes between the resistant Arabidopsis accession Bla-2 and the susceptible Kondara permit us to identify individual genes potentially related to ROS homeostasis in response to mite feeding. *BBE22*, *AO*, *GPX7* and *GSTU4* genes were also differentially induced upon the massive infestation of Arabidopsis rosette leaves with hundreds of mites for 1 h, treatment that was expected to enhance early responses at the feeding site^29^. Consistent with the expression patterns observed in the microarray experiment, we were able to replicate differential expression of these genes in Bla-2 and Kon accessions. We showed that *BBE22*, *AO* and *GSTU4* are induced early upon mite feeding, while *GPX7* was induced only after 24 h (Fig. 1). Furthermore, we previously showed that levels of H_2_O_2_ strongly increased after mite infestation in leaves from WT and *mati* plants, while they accumulated at moderate levels in the more resistant *MATI* over-expressing plants^28^. This circumstantial evidence prompted us to investigate the role of *BBE22*, *AO*, *GPX7* and *GSTU4* in the establishment of Arabidopsis responses to mite herbivory.

*BBE22*, *GPX7*, *GSTU4* and *AO* have opposing functions in the maintenance of ROS homeostasis. BBE22 belongs to the Berberine bridge enzymes (BBE), which are proteins that oxidize different molecules using FAD as a cofactor to produce H_2_O_2_^33^. In Arabidopsis, there are 28 BBE-like genes, with a physiological function mostly unknown. *BBE22* is up-regulated during lateral root initiation and upon *Pseudomonas syringae* infection^34^. *GPX7* belong to a family of thiol-based glutathione peroxidases that catalyze the reduction of H_2_O_2_ and hydroperoxides to H_2_O or alcohols using glutathione as electron donor. In addition to their detoxification activity, plant GPXs were implicated in redox signal transduction^35^. *GPX7* was linked to the establishment of the photooxidative stress tolerance and the basal resistance to *P*. *syringae* infection^36^. Plant glutathione transferases (GSTs) are thiol-based catalytic and non-catalytic proteins induced by diverse biotic and abiotic stimuli, and with an important role in protecting plants against oxidative damage^37^. *GSTU4* belongs to the plant-specific tau class of Arabidopsis glutathione transferases. *GSTU4* is induced by H_2_O_2_^38^ and was shown to have GSH-conjugating and GSH-dependent peroxidase activities^39^. Finally, ascorbate oxidases (*AO*) catalyze oxygen reduction to water using ascorbate as the electron donor. AO activity could affect the overall redox state and could contribute to creation of a hypoxic microenvironment^40^. Recently, low leaf AO activities have been associated with the resistance against aphids, associated with the more reduced redox state of the apoplast^41^. We challenged the involvement of these genes in the establishment of the Arabidopsis-mite interaction by comparing the leaf damage measured as higher surface area of chlorotic spots when mites fed on the WT, loss-of-function (for *BBE22*, *GPX7*, *GSTU4* and *AO*) and overexpression (for *BBE22*) lines. As perturbation of ROS homeostasis is expected to affect the formation of chlorotic spots, we also monitored mite fecundity as a measure of mite performance in the Arabidopsis-mite interaction (Fig. 2). Reduced expression of genes involved in H_2_O_2_ catabolism, as well as overexpression of *BBE22* (encoding a H_2_O_2_ synthesizing enzyme), resulted in increased leaf damage and increased mite fecundity. These data clearly established the involvement of these genes in the establishment of the Arabidopsis-mite interaction. However, contrary to our expectations, the expression of these genes and the effects they had on the Arabidopsis-mite interaction did not correlate with the H_2_O_2_ levels (Fig. 3).

**Figure 7:**
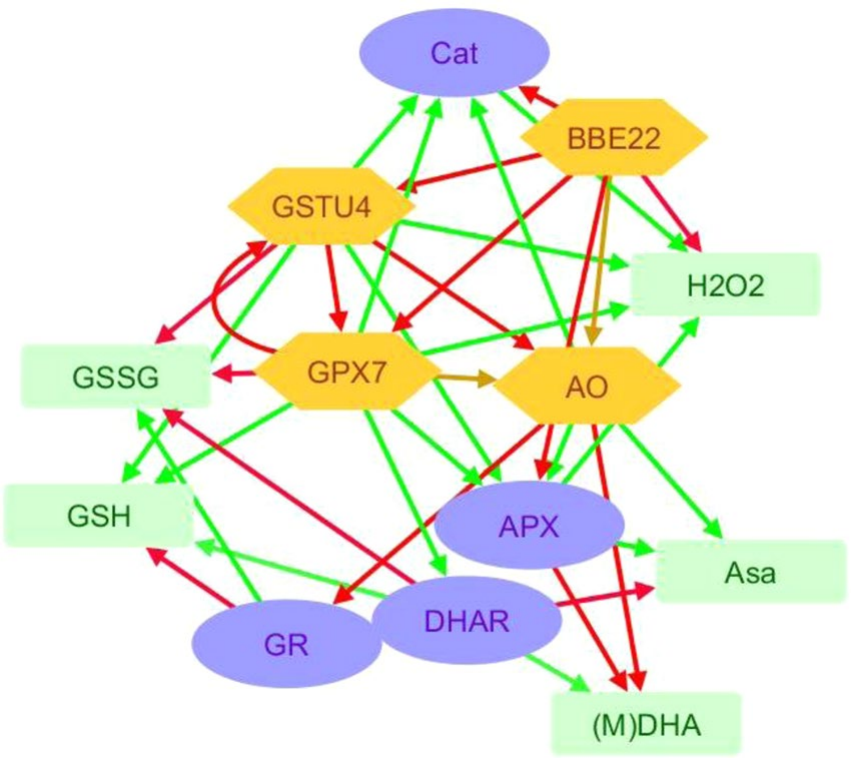
Network showing the relationships among the ROS-related features analysed. Connections involving ROS-related enzymes and enzymatic activities were inferred from the results of this work. Connections with ROS-related compounds were derived from the schematic metabolic network showed in Supplementary Fig. S1. Red arrows mark positive effects. Green arrows mark negative effects. Yellow arrows mark variable effects. Green boxes are ROS-related compounds (Asa, ascorbate; (M)DHA, (mono)dehydroascorbate; GSH, glutathione; GSSG, glutathione disulphide; H_2_O_2_, hydrogen peroxide). Orange hexagons are ROS-related enzymes (BBE, berberine-like enzyme; GST, glutathione transferase; GPX, glutathione peroxidase; AO, ascorbate oxidase). Blue ovals are enzymatic activities (Cat, catalase; GR, glutathione reductase; DHAR, dehydroascorbate reductase; APX, ascorbate peroxidase).

Several factors may contribute to this discrepancy. First, the genetic modification of any ROS-related gene was accompanied by transcriptional changes in other ROS-related genes (Fig. 5) and variations in ROS-related enzymatic activities (Fig. 4). An example of transcriptional reprogramming of ROS-related genes comes from the *BOTRYTIS-INDUCED KINASE1* (*BIK1*) gene, which was also induced in the massively mite-infested Bla-2 and Kon plants^29^, and encodes an Arabidopsis receptor-like cytoplasmic kinase involved in ROS modulation. It has been previously shown that loss of *BIK1* mutant exhibited elevated basal expression of ROS-generating and -responsive genes, but not ROS-metabolizing genes, rendering the *bik1* mutant more resistant to *M*. *persicae* than wild-type plants^42^. Figure 7 shows the complexity of the network constructed from the functional interactions found among the ROS-related enzymes, activities and compounds analyzed in this work. Multiple connections are depicted for each node, which implies a tight interrelation among the different players to deal with the dual detrimental effect/signaling role of ROS. Thus, the perturbations of the expression of the ROS-associated genes have more global effect on gene expression, modifying the final physiological output of the initial perturbation.

Second, there is a cross-talk between the ROS and hormonal signaling pathways (Fig. 7). Extensive characterization of plant-herbivore interactions has demonstrated the integration between ROS and hormonal signaling in plant defense^20,43^. ROS and hormonal pathways have been connected through glutathione status. The SA-activated transcription factor NPR1 is known to be regulated by modulation of thiol status^44,45^, and glutathione has been suggested as a factor determining basal JA gene expression^46^. Previous results in Arabidopsis plants with different levels of *MATI* expression support the link between ROS, glutathione and the hormonal response of Arabidopsis plants to spider mites^28^. As the activity of most ROS-related enzymes likely has an impact on glutathione levels, genetic manipulation of ROS-related genes could have a subsequent effect on hormonal signaling pathways. Signaling effects caused by MATI support a fine-tuning of the expression of genes involved in the hormonal network leading to final responses through the JA and SA pathways^28^. Silencing *AO*, *GPX7* and *GSTU4* lines showed an expression pattern of SA and JA related genes that mostly resemble that observed in the wild-type. However, several differences could be appreciated. AO lines have an apparent enhance of the SA pathway. GPX7 KO has a strongest induction of *PDF1*.*2* after mite treatment. GSTU4 lines showed a lower induction of *PR1* after mite infestation together variable differences in *VSP2* and *PDF1*.*2* expression. In contrast, *BBE22* had a profound effect on JA responses. Whereas loss of *BBE22* function favored MYC2 regulated responses that are marked with the *VSP2* gene, the overexpression of *BBE22* shifted the JA-responses to the antagonistic ERF1/ORA59 regulated pathway that was monitored by the expression of *PDF1*.*2* gene. Therefore, the variations in the hormonal signaling pathways would modify the resistance/susceptibility status of the T-DNA insertion lines.

Third, as H_2_O_2_ levels were quantified at the whole leaf level after 24 h of mite feeding, we cannot discard transient or compartmentalized changes in ROS signals that could affect plant response, including hormonal signaling. In fact, the interaction ROS-hormones may have complex and variable effects on plant susceptibility to the attacker. The oral secretions of *Manduca sexta* induced ROS by a rapid elicitation of the NADPH oxidase *Narboh D* expression in *Nicotiana attenuata*. Silencing *Narboh D* plants were more vulnerable to the lepidopteran *S*. *littoralis* after *M*. *sexta* elicitation but showed no difference in the induction of jasmonic acid^47^. In contrast, fatty-acid amides induced ROS in Arabidopsis and suppress insect related defenses, most likely by attenuating jasmonic acid responses. NADPH oxidase *rbohD/F* mutant plants were impaired in ROS production and were more resistant to the herbivores *Spodoptera exigua* and *Trichoplusia ni*^48^. These examples reinforce the importance of ROS in the establishment of the plant-herbivore interaction, but also point to an unpredictable final effect of manipulation of ROS-related genes.

In conclusion, the integration of ROS and ROS-related compounds and enzymes in the response of Arabidopsis to the spider mite *T*. *urticae* has been confirmed. The challenge is the elucidation of specific molecular interactions that induce plant response. Genetic manipulation of ROS-related genes permits us to deep into the pathways associated to the induction/repression of ROS enzymes. However, the complex network that includes ROS-related genes hinders the prediction of specific effects exerted by specific genes. Furthermore, additional variables, such are the participation of other enzymes/enzymatic activities in the production/elimination of ROS or the compartmentation of ROS molecules leading to transient and subcellular ROS bursts, are expected to modulate ROS-responses and should be kept in mind while interpreting the herbivore-plant ROS signals.

## Methods

### Plant material and growth conditions

*Arabidopsis thaliana* Col-0, Kondara (Kon) and Bla-2 (Bla-2) accessions (Nottingham Arabidopsis Seed Collection) were used as wild-types. *A*. *thaliana* Col-0 T-DNA insertion lines (Salk_070852, Salk _061411, Salk _023283, Salk _072007, Salk _125181, Salk _143928, Salk _118723, Salk _044265) were obtained from the Arabidopsis Biological Resource Centre (ABRC; http://www.biosci.ohio-state.edu/pcmb/Facilities/abrc/abrchome.htm) through the European Arabidopsis Stock Centre (NASC; http://arabidopsis.info/BasicForm/). T-DNA insertion and homozygous status of the Salk lines were validated by PCR. For soil growth, peat moss and vermiculite (2:1 v/v) was used. Sterilized seeds were stratified in the dark at 4 °C for 5 days. Plants were then grown in growth chambers (Sanyo MLR-350-H) under controlled conditions (23 °C ± 1 °C, >70% relative humidity, a 16 h/8 h day/night photoperiod, and a light intensity of 138 μmol m^−2^ s^−1^).

### Gene expression analyses by real time PCR (RT-qPCR)

*A*. *thaliana* rosettes from Col-0, Bla-2 and Kon accessions were sampled after different times of mite infestation (1, 3, 6, 12 and 24 h). *A*. *thaliana* Col-0 flowers, roots, siliques, leaves from stems, and 1, 2 and 3 week-old rosettes were also collected. In addition, Arabidopsis entire plants from selected T-DNA insertion lines and from the non-transformed Col-0 controls were collected. Total RNA was extracted as previously described^49^ and reverse transcribed using Revert AidTM H Minus First Strand cDNA Synthesis Kit (Fermentas). RT-qPCR was performed for triplicate samples as previously described^30^ using a SYBR Green detection system (Roche) and the CFX Manager Software 2.0 (Bio-Rad). Ubiquitin was used as housekeeping gene for normalization. Gene expression was referred as relative expression levels (2^−dCt^) or fold change (2^−ddCt^)^50^. Specific primers were designed through the PRIMER 3 program (http://bioinfo.ut.ee/primer3-0.4.0/). Primer sequences are indicated in Supplementary Table S2.

### Spider mite maintenance and fitness analyses

A colony of *T*. *urticae*, London strain (Acari: Tetranychidae) provided by Dr. Miodrag Grbic (UWO, Canada), was reared on beans (*Phaseolus vulgaris*) and maintained in growth chambers (Sanyo MLR-350-H) at 25 °C ± 1 °C, >70% relative humidity and a 16 h/8 h day/night photoperiod. Mites were synchronized by inoculating 100 adult females (random age) on one leaf of bean confined in a closed system under water-soaked cotton. After one day, adult females were removed, and 10 days after, population on the leaf was synchronized. Spider mite fecundity was recorded on T-DNA insertion lines and non-transformed Col-0 controls. *T*. *urticae* fecundity assay was performed on detached leaves from 3 week-old plants. The newest emerged leaf (about 1 cm long) from each plant was placed in special dishes and infested with 12 synchronized adult females. After 36 h of infestation, the number of eggs was counted. Eight replicates were used for each plant genotype.

### Plant damage determination

Quantification of plant damage after spider mite feeding was done on Arabidopsis entire plants from selected T-DNA insertion lines and non-transformed Col-0 controls. Three week-old plants were infected with 20 *T*. *urticae* female adults per plant. After 4 days of feeding, leaf damage was assessed by scanning the entire rosette using a hp scanjet (HP Scanjet 5590 Digital Flatbed Scanner series), as previously described^27^. Leaf damage was calculated in mm^2^, using Adobe Photoshop CS software. Six replicates were used for each genotype.

### H_2_O_2_ determination in leaf extracts

Three week-old Arabidopsis plants from T-DNA insertion lines and non-transformed Col-0 controls were infested with 20 mites and incubated for 24 h. The H_2_O_2_ concentration of crude extracts was determined by spectrofluorometry, basically as previously described^51^. All operations were performed at 0–4 °C. Leaves (0.1 g) from rosettes were homogenized in 300 µL 25 mM H_2_S0_4_ and supernatants collected by centrifugation at 15,890 g for 5 min. The pH of leaf extracts was adjusted to 7.0 with NaOH and these extracts were used to measure the H_2_O_2_ concentration. The reaction mixtures (300 µl) contained 50 mM Hepes buffer, pH 7.6, 50 mM homovanillic acid and 10 µL of sample. The reaction was started by adding 4 µM horseradish peroxidase and the fluorescence produced was measured in a Varioskan® LUX (Thermo Fisher Scientific) microplate reader at excitation and emission wavelengths of 315 and 425 nm, respectively. The H_2_O_2_ concentration was determined from a calibration curve of H_2_O_2_ (Sigma) in the range 0.1–1000 µM. Three replicates were done by genotype and treatment.

The accumulation of H_2_O_2_ was visualized using the 3,3-diaminobenzidine tetrachloridehydrate (DAB) substrate (Sigma-Aldrich) which produces a brown precipitate after oxidation in the presence of H_2_O_2_^52^. Col-0 Arabidopsis leaf disks (1 cm diameter) from T-DNA insertion lines and the non-transformed Col-0 controls were infested with 10 mites and incubated for 24 h. Infested and non-infested control disks were stained with DAB^53^ and observed under a Leica fluorescence stereoscope. DAB staining specificity was confirmed in presence of the H_2_O_2_ scavenger, ascorbic acid (10 mM).

### Antioxidant enzyme activity measurements

Three week-old Arabidopsis plants from T-DNA insertion lines and the non-transformed Col-0 controls were infested with 20 mites and incubated for 24 h. Ascorbate–glutathione recycling enzymes were extracted from rosette leaves into a pre-cooled extraction buffer consisting of 1.5 ml of 0.1 M NaH_2_PO_4_, 1 mM EDTA (pH 7.5), 1% (w/v) insoluble PVP, 1 mM ascorbate. 1 M ascorbate was freshly prepared and added to the extraction medium with an approximated ratio of 1.5 ml/150 mg FW. The homogenate was centrifuged at 14,000 g for 10 min at 4 °C and the supernatant was used immediately for enzyme activities. All assays were performed in a final volume of 0.2 ml per well in a UV-microplate at 25 °C. Samples, controls and blanks were analyzed in triplicate. The microplate reader was a Varioskan® LUX (Thermo Fisher Scientific) spectrophotometer. The SkanIt RE 4.0 software was used to check the reader and to analyze enzymatic reactions. Ascorbate peroxidase (APX, EC 1.11.1.11), dehydroascorbate reductase (DHAR, EC 1.8.5.1) and glutathione reductase (GR, EC 1.6.4.2) activities were quantified as previously described^54^ with slight modifications. Catalase (CAT, EC 1.11.1.6) activity was measured by determining the degree of H_2_O_2_ decomposition at 240 nm for 2 min^55^. The reaction buffer consisted of 0.1 M NaH_2_PO_4_, 1 mM EDTA (pH 7.5). Then, 2 M H_2_O_2_ was added to each well (40 mM) and absorbance was recorded. The reaction was initiated by the addition of 10 µl of crude extract. All specific activities were calculated using the extinction coefficients and referring the results to the milligrams of protein in the extract (determined according to the method of Bradford^56^, with bovine serum albumin as standard). For APX, (ɛ_290_ = 2.8 mM^−1^ cm^−1^), the results indicate the µmol of ascorbate oxidized per minute. Cat activity (ɛ_240_ = 40 M^−1^ cm^−1^) is expressed as H_2_O_2_ mmol removed in a minute. For DHAR (ɛ_265_ = 14 mM^−1^ cm^−1^), results represent mmol of DHA reduced per minute. In the case of GR (ɛ_340_ = 6.22 mM^−1^ cm^−1^), data show mmol of NADPH oxidized in a minute. At least six replicates were done by genotype and treatment.

### Bioinformatics and statistical analyses

Structural information on Arabidopsis genes was retrieved from the Arabidopsis Information Portal (Araport, https://www.araport.org/) using the Araport11 annotation^57^. Protein domains predictions were performed in Pfam database^58^ using Pfam 31.0 version (http://pfam.xfam.org/). Network of ROS-related actors was constructed in Cytoscape^59^ using the 3.6.0 version (http://www.cytoscape.org/).

Statistical analysis was performed using One-Way ANOVA for gene expression in Arabidopsis accessions, damage analysis and spider mite bioassays. Two-Way ANOVA was used for H_2_O_2_ accumulation. For the enzymatic activities and gene expression studies in T-DNA lines two analysis were performed, One-Way ANOVA to compare the effect of the genotype within basal or infested conditions and T-Student to compare within each genotype the effect of the infestation. ANOVA analyses were followed by the Student-Newman-Keuls multiple comparison test. In figures, significant differences (P < 0.05) are reported with different letters or asterisks depending on the analysis performed.

## Electronic supplementary material

Supplementary material
