# Supplementary material for: Arabidopsis response to the spider mite Tetranychus urticae depends on the regulation of reactive oxygen species homeostasis
Source: Sci Rep. 2018 Jun 21;8:9432. doi: 10.1038/s41598-018-27904-1 (PMC6013483; doi:10.1038/s41598-018-27904-1)
Supplement: Supplementary file 1 — Supplementary material [file 41598_2018_27904_MOESM1_ESM.pdf]

Supplementary Information

Arabidopsis response to the spider mite *Tetranychus urticae* depends on the regulation of reactive oxygen species homeostasis

M Estrella Santamaría, Ana Arnaiz, Blanca Velasco-Arroyo, Vojislava Grbic, Isabel Diaz, Manuel Martinez

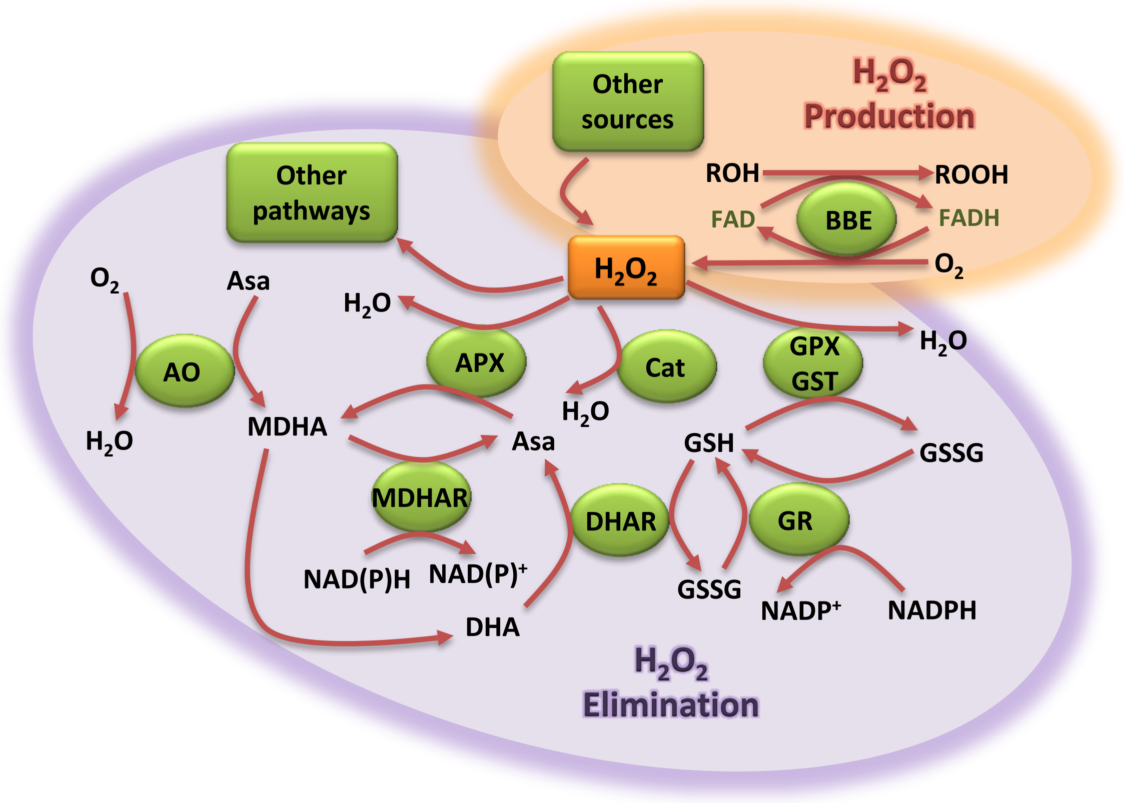

Supplementary Figure 1. Schematic representation of the metabolic pathways leading to H_2O_2 production and elimination. For clarity, a simplified metabolic network was done with a special focus on the molecules analysed in this work. H_2O_2, hydrogen peroxide; Asa, ascorbate; (M)DHA, (mono)dehydroascorbate; GSH, glutathione; GSSG, glutathione disulphide; BBE, berberine-like enzyme; GST, glutathione transferase; GPX, glutathione peroxidase; AO, ascorbate oxidase; Cat, catalase; GR, glutathione reductase; (M)DHAR, (mono)dehydroascorbate reductase; APX, ascorbate peroxidase; NAD(P), nicotinamide adenine dinucleotide (phosphate); FAD, flavin adenine dinucleotide.

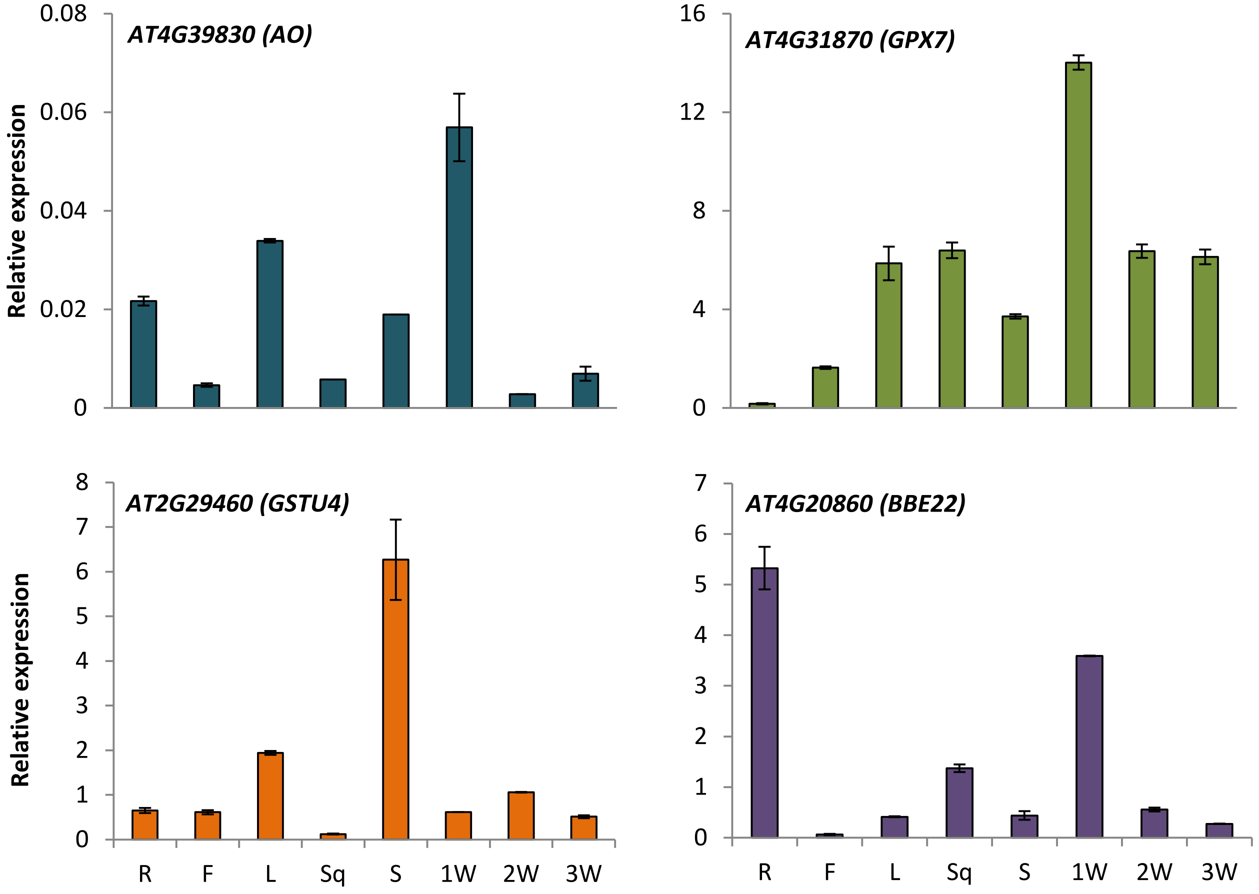

Supplementary Figure 2. Gene expression in plant tissues. Relative gene expression levels (2^-dCt) of *AT4G39830*, *AT4G31870*, *AT2G29460* and *AT4G20860* in different Arabidopsis tissues. Roots (R), flowers (F), leaves from stem (L), siliques (Sq), seeds (S) and rosettes from 1, 2 and 3 week-old (1W, 2W, 3W). Ubiquitin was used to normalize gene expression.

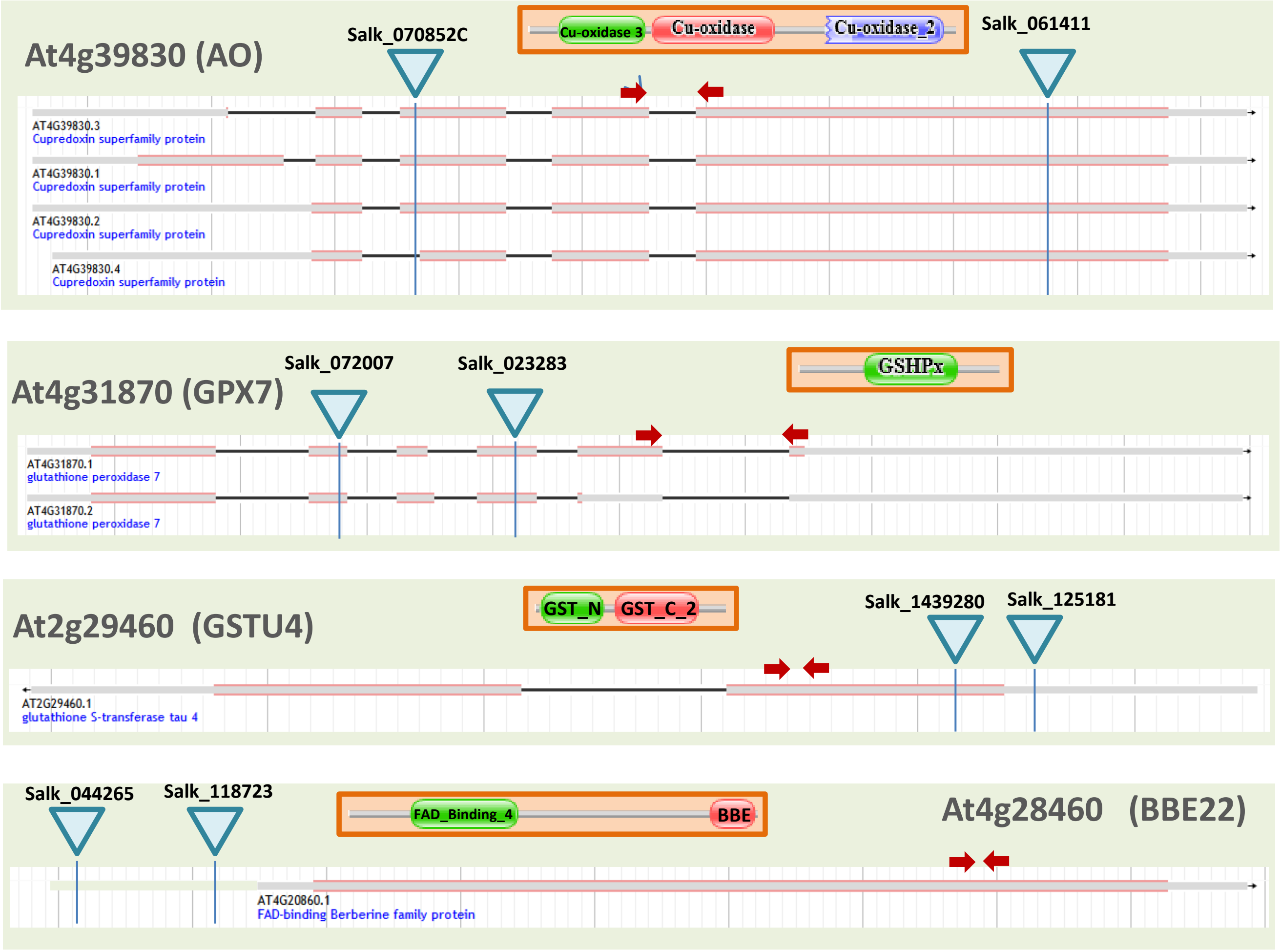

Supplementary Figure 3. Structural information of selected Arabidopsis genes. A schematic representation of protein domains is shown above the genomic structure of each gene (exons, grey boxes; introns, black lines; coding region, grey-red boxes). Red arrows mark the position of oligonucleotides used in RT-qPCR experiments. Blue triangles point to the gene location in which the T-DNA was inserted.

Supplemental Table 1. Fold change of the expression of ROS-related genes in T-DNA insertion lines.

T-DNA line	Fold change ± standard error			
	AO	*GPX7*	*GSTU4*	*BBE22*
**AO KD1**	0.40 ± 0.02	1.12 ± 0.04	1.33 ± 0.17	2.69 ± 0.39
**AO KD2**	0.65 ± 0.04	0.83 ± 0.02	1.10 ± 0.24	1.98 ± 0.04
**GPX7 KD**	0.53 ± 0.01	0.68 ± 0.01	1.02 ± 0.14	2.16 ± 0.37
**GPX7 KO**	1.85 ± 0.13	0.00 ± 0.00	0.23 ± 0.04	1.17 ± 0.10
**GSTU4 KD1**	0.69 ± 0.10	0.67 ± 0.02	0.11 ± 0.07	1.30 ± 0.08
**GSTU4 KD2**	0.42 ± 0.03	0.88 ± 0.01	0.09 ± 0.05	1.31 ± 0.10
**BBE22 KO**	0.17 ± 0.03	0.55 ± 0.01	0.74 ± 0.21	0.03 ± 0.01
**BBE22 OE**	0.49 ± 0.02	0.82 ± 0.06	2.11 ± 0.19	29.95 ± 2.82

Supplemental Table 2. Sequences of used oligonucleotides (F: forward; R: reverse).

Gene	Primer name	Sequence (5´ → 3´)
Ubiquitin	UBQ-F	GCTCTTATCAAAGGACCTTCGG
	UBQ-R	CGAACTTGAGGAGGTTGCAAAG
AT4G39830	AO-F	TTCCCGGAAAGACATACAGG
	AO-R	TCCGTCTGCTTCAACAACTG
AT4G31870	GPX7-F	AGGGCAAAGTTGTCGAGAGA
	GPX7-R	TTAAGCCGCAAGCAACTTCT
AT2G29460	GSTU4-F	AGATTTCTTCGGTGGCAAAA
	GSTU4-R	ATTCCCATACCTTCCCAACC
AT4G20860	BBE22-F	TTTCTAAATCGCCGAGAGGA
	BBE22-R	TTGCATCTTCGAAACTCGTG
AT1G64280	NPR1-F	TGCATCAGAAGCAACTTTGG
	NPR1-R	GGCCTTTGAGAGAATGCTTG
AT2G14610	PR1-F	TCAGTGAGACTCGGATGTGC
	PR1-R	CGTTCACATAATTCCCACGA
AT1G32640	MYC2-F	TCCGAGTCCGGTTCATTCT
	MYC2-R	TCTCGGGAGAAAGTGTTATTGAA
AT5G24770	VSP2-F	ATGCCAAAGGACTTGCCCTA
	VSP2-R	CGGGTCGGTCTTCTCTGTTC
AT5G44420	PDF1.2-F	GTTCTCTTTGCTGCTTTCGAC
	PDF1.2-R	GCAAACCCCTGACCATGT
